# The Problems and Design of a Neck Dummy

**DOI:** 10.3390/biomimetics9110661

**Published:** 2024-10-31

**Authors:** Christopher René Torres San Miguel, José Antonio Perez Valdez, Marco Ceccarelli, Matteo Russo

**Affiliations:** 1 Instituto Politécnico Nacional, Escuela Superior de Ingeniería Mecánica y Eléctrica, Nacional Unidad Zacatenco, Sección de Estudios de Posgrado e Investigación, Ciudad de México 07738, Mexico; ctorress@ipn.mx (C.R.T.S.M.); jperezv1404@alumno.ipn.mx (J.A.P.V.); 2 LARM2: Laboratory of Robot Mechatronics, University of Rome “Tor Vergata”, 00133 Rome, Italy; matteo.russo@uniroma2.eu

**Keywords:** biomechanics, injury risk evaluation, dummies, neck dummies, testing

## Abstract

This paper addresses the biomechanical requirements and design of a neck dummy for assessing neck injury risks. The need for an accurate biomechanical representation of the human neck in crash tests is highlighted, emphasizing the importance of replicating the neck’s response to impacts. Existing neck dummies are reviewed to assess their similarity to human neck biomechanics, revealing several limitations. To address these gaps, a novel prototype is proposed to mimic the joint between two vertebrae using elastic elements to replicate the behavior of the intervertebral disc. The performance of the neck dummy is evaluated through experimental testing, using IMU and force sensors to monitor its response to perturbations from impacts. The reported results demonstrate that the prototype effectively simulates the intervertebral movement, offering an approach for more accurate injury assessments in crash testing. Concluding remarks suggest the potential of this design to improve the reliability of neck injury assessments in automotive safety research.

## 1. Introduction

Automotive accidents are one of the leading causes of death around the world, with 2.5% of total deaths, and the fourth leading cause of non-fatal, unintentional injuries accordingly to the World Health Organization [1]. The neck is the most commonly injured part, with 30.1% among non-fatally injured persons [2]. The neck usually presents different injuries according to the type of impact to which it is subjected, among which whiplash-associated disorder (WAD) is the main and most studied injury in the neck. The mechanism of injury in whiplash trauma takes 50–75 ms for the neck to curve into an “S” shape from the neutral posture. The intervertebral joints in the lower cervical spine display hyper-extension that goes beyond their natural bounds during this first stage, and as a consequence, the entire spine extends, and the intervertebral discs suffer damage. During the latter phase, the whole cervical spine experiences extension, but the articulations do not move past their physiological bounds [3]. This kind of injury is associated with long-term complications and damage in the neck soft tissue [4,5] with an estimated cost per year of USD 2.7 billion in the U.S.A. and 3 billion in the U.K. [6]. Although the exact mechanism of whiplash injury is not yet fully understood, several authors agree that factors such as the impact velocity, direction of force, and individual biomechanics of occupants play crucial roles in the severity of the injury [7,8,9].

In addition to WAD injuries, which are the result of rear-end collisions, there are injuries produced by frontal and lateral crashes. One-third of total neck injuries occur in frontal collisions [10]. During frontal collisions, occupants may report the initial whiplash symptoms and may suffer permanent disability after one year from the crash. Many authors have studied neck injuries in frontal collisions [11,12] analyzing the influence of the curve of acceleration on the risk of long-term disability in the neck.

In order to characterize the behavior of the neck under impact conditions, researchers use human volunteers [13], postmortem human bodies [12], numerical simulations [11], and anthropomorphic test devices (ATDs) [14,15,16]. The results from numerical simulation are useful tools to analyze the response of a simulated neck and to verify the mechanical characteristics useful for the development of impact dummies. In such studies, the response of the cervical spine can be defined as an impulse, as in [17], when simulating a rear impact on a skull–neck model. On the other hand, the displacement response can be characterized according to time by simplifying the simulation to one plane and analyzing the trajectory of the vertebrae, as in [18]. The results of these studies provide a point of reference for the evaluation of the kinematic behavior of physical models in experimental tests.

Currently, anthropomorphic dummies are used in crash testing to simulate distinct parts of the human body in terms of mass, size, and width. Their job is to capture data on displacements, accelerations, and forces occurring on specific regions of the dummy’s body during a collision. The data from the dummy demonstrate the extent of harm or human survival in a vehicle collision. The similarity in behavior between ATDs and the human body is crucial to accurately assess injury rates and to improve the development of vehicle safety systems [19]. For an ATD to correctly replicate the behavior of the human neck in an impact, the dummy neck must closely meet the dimensions, mass, range of motion, and structure of the cervical spine, such as the vertebrae, the soft tissue, and the mass of the head [20,21,22]. In addition, the mechanical properties of the intervertebral discs (IVDs) play an essential role in the kinematic behavior of the neck, being a significant source of injury in certain types of impact.

Unfortunately, a dummy is meant to be utilized in a specific crash test with a specified crash speed. This means that, despite the dummy’s exact biomechanic similarity to the human body, dummy makers will not approve the use of the same dummy for multiple crash testing. Furthermore, the market lacks a dummy that is devoted to low-speed crash tests.

The development of a dummy neck that accurately replicates the human neck under impact conditions is essential to advance vehicle safety. Such a neck dummy considers not only the dimensions and geometry of the cervical vertebrae but also the integration of systems that help in replicating neck flexibility. Additionally, it must be provided with a system to measure accelerations and forces at key points to assess relevant injury rates.

The aim of this paper is to present a design of a neck dummy that faithfully reproduces the internal and external structures of a human neck, to allow a more accurate assessment of injury risks. Analyzing the reaction force in the intervertebral disc and the movement of each cervical vertebra, the proposed neck dummy will contribute to developing more reliable restraint systems that can be useful in reducing the incidence and severity of neck injuries in car crashes. In the literature mentioned above, existing dummies allow for the evaluation of a neck injury index using sensors at limited points on the dummy. Conversely, in the proposed prototype, an extensive data acquisition system is proposed to better characterize the response of the cervical spine to an impact on each cervical vertebra. The research and development of the proposed prototype will not only improve the reliability of ATDs but will also provide crucial data for the continuous improvement in road safety measures.

## 2. Materials and Methods

To design a neck dummy, it is necessary to take into account aspects of the anatomy of the neck and the biomechanics of the cervical spine. Biomechanical requirements need to be considered in order to replicate the structure, geometry, and mobility of the cervical spine. They are described in the following section as those considered for the proposed design.

### 2.1. Human Neck

The cervical spine constitutes a structure that supports the head and moves and orients it in three-dimensional space. The muscles execute the movement of the head, but the type of possible movements depends on the shape and structure of the cervical vertebrae and the interaction between them. The kinematics of the cervical spine is therefore based on the geometry of the bones, the whole structure of the spine, and the intervertebral discs which are the joints between the vertebrae [23].

**Figure 1:**
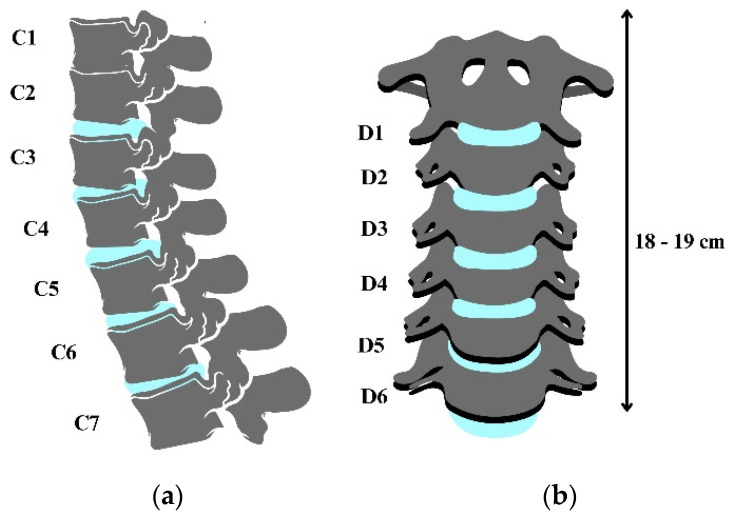
Anatomy of a cervical spine: (**a**) vertebrae (C1 to C7); (**b**) intervertebral discs (D1 to D6).

A cervical spine is made of 7 vertebrae, as shown in Figure 1, from vertebrae C7 to C1. The total length of a neck varies from 18 to 19 cm in men; ¼ of this length is represented by the intervertebral discs [24]. Usually, the spine functions as a unit. The kinematics of the neck is analyzed by studying the motion of the head relative to the body. According to Ramieri et al. [25], the kinematics of the cervical spine can be analyzed from the point of view of a functional spine unit, which consists of two adjacent vertebrae and the soft tissue that connects them.

**Figure 2:**
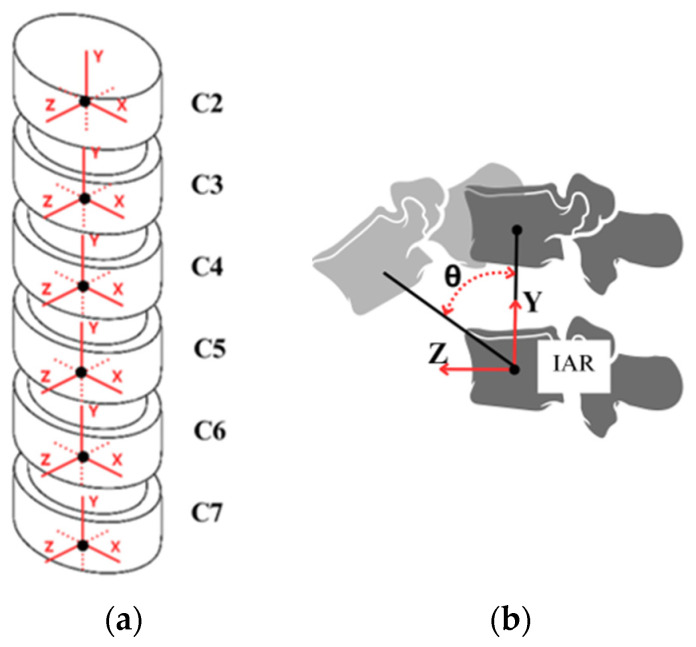
Degrees of freedom of the cervical spine: (**a**) reference frames; (**b**) instant axis of rotation (IAR) between two vertebrae in sagittal plane.

Due to the connection of a functional spine unit, when a force is applied at some point of the cervical spine, a torque is produced and causes a rotation around an axis, which can be defined as the “instantaneous axis of rotation” (IAR) between vertebrae, as shown in Figure 2b. Based on the cartesian coordinates (x, y, z) shown in Figure 2a, 12 possible movements in the cervical spine can be considered in relation to the IARs with three translations and three rotations along and around each IAR. This means that there are 6 degrees of freedom in each two-vertebra unit due to the flexible joint by the intervertebral disc. The 6 degrees of freedom of the relative motion between vertebrae refer to the possible movements around and along the axes of a reference frame xyz in Figure 2a, while the main rotation in the sagittal plane is illustrated in Figure 2b.

The cervical spine has the possibility to move in flexion, which is the forward rotation of the head and neck, in extension, which is the backward rotation of the head and neck, in lateral flexion, and in axial rotation. From the point of view of kinematic characteristics, the cervical spine can be divided into three main sections, the craniovertebral junction (cranium to C1), the upper cervical spine (C1–C2), and the lower cervical spine (C3–C7), as per different anatomy and kinematics [22]. The junction of the C1 vertebra (Atlas) with the occipital section of the cranium allows the flexion and extension of the neck with a range of approximately 75° due to the superior articular surfaces of C1 concave and the cranium convex condyles. The upper cervical spine is responsible for 40% of the axial rotation of the neck with an average range of 45°. This displacement is allowed by the geometry and fixation between C1 and C2, where vertebra C1 rotates around the odontoid structure in vertebra C2 [23]. The characteristic values are listed in Table 1.

The flexible behavior of the neck is due to the mechanical properties of the intervertebral disc. The discs support compressive forces in association with other tissues. Under normal physiological conditions, the weight of the head affects the C2 to T1 disc with compression. Thus, the fundamental functional mechanical role of intervertebral discs is to respond to compressive loading. These characteristics are summarized in terms of elastic modulus and stiffness by Yoganandan et al. [27], as shown in Table 2.

In addition to the characteristics of the cervical spine, it is important to consider the mass and inertia of the head, since the importance of head-on-neck kinematics is highlighted in several studies. The accurate representation of head physical properties is crucial for assessing head–neck dynamics and injury metrics [28]. The dimensions and physical properties of the head are based on those used for the development of impact dummies, as, for example, in the Hybrid III dummy, with dimensional statistics such as those conducted by Yoganandan et al. [29] as listed in Table 3.

**Figure 3:**
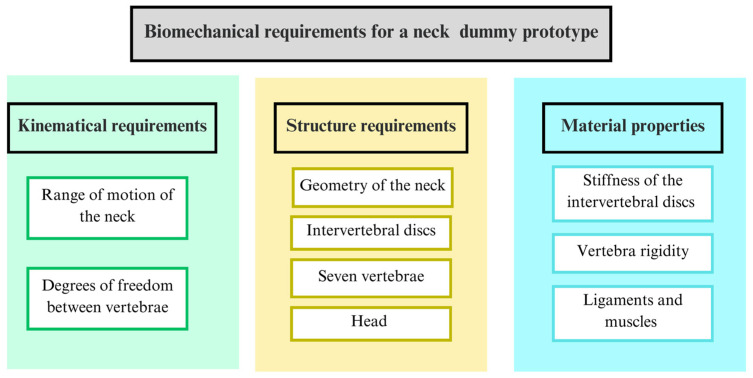
A summary of biomechanical requirements for neck dummy design.

A summary of the biomechanical aspects as requirements for a neck dummy design is reported in Figure 3 indicating the main components and characteristics to be replicated for the human cervical spine biomechanics.

### 2.2. Problems with Neck Dummies

The main problem for a functional neck dummy is to replicate the dimensions and geometry of the neck and head correctly. The characteristics of its components must also allow it to replicate the kinematic and mechanical behavior of the neck in order to offer reliable test results. In addition, a neck dummy must have sensors to record the data from the simulated accident and to be able to evaluate the probability of injury in a human.

Different neck dummies have been developed over time to solve the problem of replicating the kinematics of the neck. Currently, neck dummies are divided into three groups depending on the impact type: frontal impact, rear impact, and side impact. Dummies in these groups have specific characteristics that make them suitable for the type of impact of interest. Among the front impact dummies, there is the Hybrid III, which is the most widely used neck dummy nowadays, which was developed in 1977 by General Motors [30]. In order to replicate the dimensions and behavior of an average adult male, the National Highway Safety Administration (NHTSA) has also developed an ATD neck dummy with anthropomorphic features called THOR [31], whose parts are intended to closely replicate the human body and its mechanical and geometrical characteristics. The similarity between the biomechanical behavior of these two neck dummies and tests with humans have been evaluated by Albert et al. [32], where these two neck dummies were subjected to flexion, lateral flexion, and rotation forces. The evaluation shows the lack of similarity of the Hybrid III neck with respect to human neck behavior in most cases, and the THOR 50M results were closer to the human neck response but with a lack of similarity in the frontal flexion and torsion moment.

For lateral tests, in 1997, the international standards organization (ISO) initiated the development of a neck dummy, called WorldSID [33], which was based on a global 50th percentile male anatomy. This dummy is the one currently used by the New Car Assessment Program (NCAP) for the different lateral collision tests due to its reproducibility, durability, and sensitivity, which are better when compared to the other satisfactory dummies.

The main objective with rear crash tests is to evaluate whiplash-associated disorder (WAD). The BioRID II ATD is used successfully for this case since it features an articulated spine that more closely mimics human kinematics [34]. This dummy has a full vertebral spine, with 33 vertebrae from the lumbar section to the cervical section, which is shaped with the proper curvature of the cervical spine. This design allows the evaluation of the displacement of each vertebra during crash tests with the main mechanical aspects of whiplash injury. Studies show its reliability and biomechanical similarity to human body behavior.

**Figure 4:**
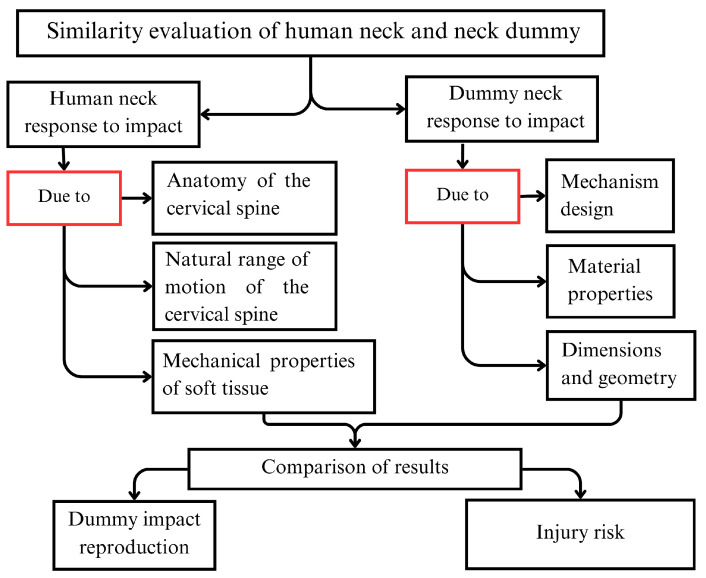
A flowchart to evaluate the similarity of a neck dummy to the human neck.

To assess the response of a neck dummy, prototype test data should be compared to human or cadaver test responses in order to evaluate the similarity between the prototype response and human neck behavior. The closer the response of a neck dummy is to that of the human, the more accurate the calculation of injury risk. The considerations for this comparison are summarized in the flow chart in Figure 4.

### 2.3. Neck Injury Indices

Injury criteria correlate the probability of trauma to mechanical parameters which can be measured using instrumented dummies or cadavers in crash tests. Without injury criteria, the intensity of trauma in a staged test or accident reconstruction cannot be determined, as pointed out in [35]. In the following we examine the main indices that have been formulated for the evaluation of injury risk considering neck behavior:

- Neck injury criteria $N_{ij}$.

The NHTSA proposed this injury index to assess severe injuries in frontal impacts, including cases with airbag expansion and impact conditions with high velocity variation [36]. The FMVSS No. 208 includes individual tolerance limits for compression, tension, shear, flexion moment, and extension moment. The tolerance values are based on tests with volunteers, cadavers, and dummies. The main concepts for the *Nij* suggest the consideration of a combination of axial forces with moments for a composite injury rate. The proposed *Nij* implies a linear combination of axial loads and bending moment, both normalized by critical intercept values in the form

(1) $$N_{ij}=\frac{F_{z}}{F_{int}}+\frac{M_{y}}{M_{int}}$$

where $F_{z}$ and $M_{y}$ are the axial loads and sagittal bending moment, respectively, and $F_{int}$ and $M_{int}$ indicate the critical values established by the NHTSA.

- Neck injury criterion NIC.

According to ECE regulation No.94, the risk of injury depends on the duration of the load and the magnitude of the load. Based on the fact that the pressure gradient caused by a sudden change in fluid flow within the next fluid compartments of the cervical spine is related to neck injury, Schmitt K. et al. [37] proposed the neck injury criterion NIC. The criterion is given by the relationship that predicts injury caused by pressure gradients between the acceleration of the posterior anterior direction of the center of gravity of the head relative to the first thoracic vertebra and the velocity, which can be expressed as

(2) $$NIC=(0.2\times a_{rel})+v_{rel}^{2}$$

where $a_{rel}$ and $v_{rel}$ are the relative acceleration and speed against thoracic vertebrae.

- Protection of neck injury criterion $N_{km}$.

The neck protection criterion $N_{km}$ is based on the assumption that a linear combination of loads and moments describes the relevant loads on the neck well. However, regarding the injuries possible in rear impact collisions, shear forces in the sagittal plane in the ligament of the axial loads are the loads that are considered critical. This combination of the sagittal and shear moment describes the constriction that is usually found in the cervical spine during the formation of the S-position in the case of whiplash, so the criterion is formulated as [38]

(3) $$N_{km}\left(t\right)=\frac{M_{y}\left(t\right)}{M_{int}}+\frac{F_{x}\left(t\right)}{F_{int}}$$

where $F_{x}\left(t\right)$ and $M_{y}\left(t\right)$ are the shear forces and bending moment that are measured by a load cell at the top of the neck, and $F_{int}$ and $M_{int}$ represent the critical values used for normalization.

- Neck–head injury criterion NHIC.

Garrosa et al. [39] developed a new injury criterion taking into account parameters such as the mass of the occupant, the mass of the vehicle, the vehicle speed, and the accelerations and displacement of the head and neck in the expression

(4) $$NHIC=\frac{\left(m_{v}+m\right)a_{vmax}}{m({v_{v})}^{2}}\times \frac{a_{hmax}}{a_{nmax}}\left(\left(X_{hi}-X_{hf}\right)+\left(X_{ti}-X_{tf}\right)\right)\times 1000$$

where $m_{v}$ is the vehicle weight, m is the occupant weight, $a_{vmax}$ is the maximum vehicle acceleration, $v_{v}$ is the vehicle speed at the moment of the experiment, $a_{hmax}$ is the maximum head acceleration, $a_{nmax}$ is the maximum neck acceleration, $X_{hi}$ is the initial distance of the head from a reference point, $X_{hf}$ is the final distance of the head to the final distance, $X_{ti}$ is the initial distance of the torso, and $X_{tf}$ is the final distance of torso. This injury criterion explicitly considers neck motion as influential in the injury risk evaluation.

Each of the above injury criteria defines a range within which the neck does not suffer any injury or a critical value with a probability of injury. These critical values are summarized in Table 4.

### 2.4. Design of a New Prototype

**Figure 5:**
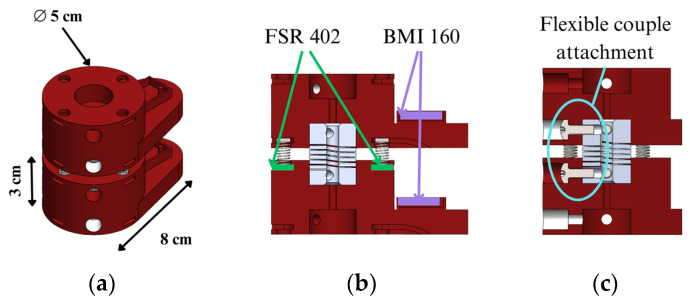
Design of a two-vertebra unit assembly: (**a**) isometric view; (**b**) cut view on the lateral plane; (**c**) 45-degree cut view.

This section describes how the proposed neck dummy is designed, referring to a two-vertebra unit. The vertebrae are designed in a two-vertebra unit, taking into account only the vertebral body and the junction between vertebrae. Each vertebra is sized with a height of 3 cm and a diameter of 5 cm, as shown in Figure 5a. In the upper and lower faces of vertebrae, there are five holes, with a central one to fix a flexible aluminum coupling simulating the intervertebral disc. Four more holes are arranged at 90 degrees to each other, in which metal springs and FSR402 resistive force sensors are housed. From the bottom of this, there is a protrusion that replicates the vertebra shape with a depth of 8 cm. This section houses a BMI160 IMU sensor on the surface to monitor the acceleration and displacement of the vertebra. The location of these sensors can be seen in Figure 5b. The joining of the two vertebrae is carried out by an aluminum coupling through two screws from the front of each vertebra, as shown in Figure 5c. This leaves a separation between each vertebra of 1 cm.

The location of the force sensors is designed to acquire the force and moments when the neck is subjected to flexion, extension, and lateral bending. On the other hand, the acceleration sensor on the vertebra allows the acquisition of the acceleration of the vertebra and its angular velocity with respect to three axes. With these results, the magnitude of the acceleration, angular displacement, and tangential velocity are computed to calculate the injury index and the response of the prototype to any perturbation.

**Figure 6:**
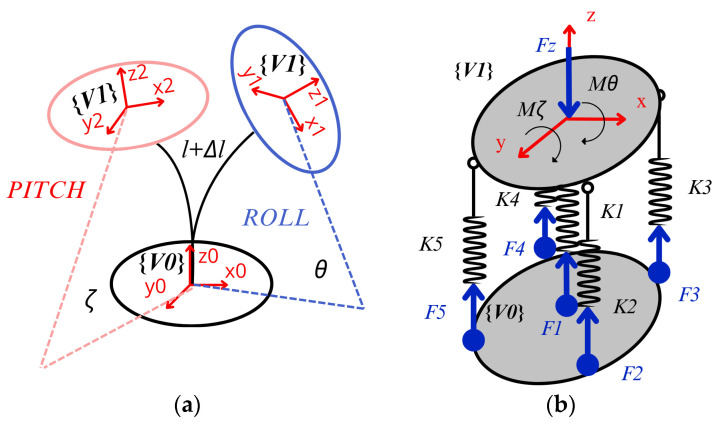
Functional model for vertebra unit: (**a**) main motion parameters; (**b**) free body diagram.

The functional modes of the assembly in Figure 5 are described in Figure 6. The vertebra unit has two main rotational degrees of freedom due to the flexibility of the coupling represented by angles ζ and θ in Figure 6a and one translational degree of freedom with elongation Δl along z axis that is limited by the stiffness of the elastic couple and the attached metal springs, as Figure 6b shows.

From Figure 6, the three degrees of freedom can be described using the following parameters considering reference frames fixed on vertebra bodies:

- Lateral bending angle θ (Roll), which is defined as the angle between the x0z0 plane in the lower vertebrae {V0} and the x1z1 plane in the upper vertebrae {V1};
- Flexion–extension angle ζ (Pitch), which is defined as the angle between the y0z0 plane in the lower vertebrae {V0} and the y2z2 plane in the upper vertebrae {V1};
- Axial elongation and compression Δl, which is defined as the variation in distance between two vertebrae along the z axis.

To characterize the intervertebral disc, the stiffness of the aluminum flexible coupling and the springs are considered with compression module $k_{1}$ and a bending stiffness $k_{b}$ from material properties. The stiffness values of elastic springs are represented by $k_{2},k_{3},k_{4},k_{5}$ in Figure 6b, with the corresponding elastic forces $F_{i}(i=1,2,3,4,5)$.

### 2.5. Testing Design

**Figure 7:**
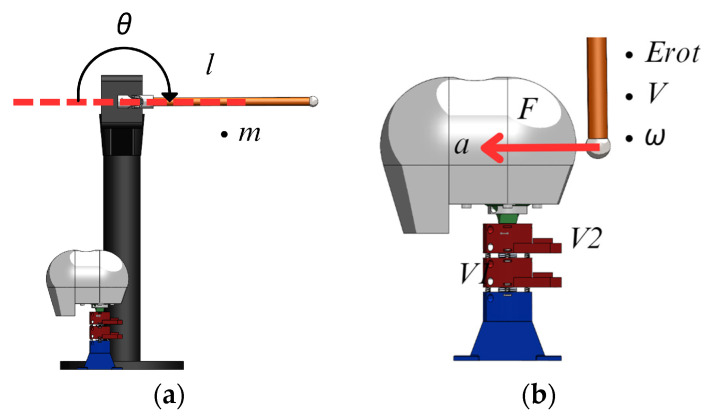
Testing layout: (**a**) initial position of the pendulum; (**b**) impact conditions on the neck.

Tests of the built prototype are carried out simulating an impact using a pendulum, as shown in Figure 7, with a 38.6 cm steel tube of 2 cm diameter, a mass of 225 g, holding a metallic sphere with a diameter of 0.34 cm and a mass of 82 g at the edge. In the initial condition of the test, the pendulum is placed horizontally. When the pendulum reaches vertical posture at impact, it has an angular speed of 6.9 rad/s with a rotational kinetic energy $E_{rot}$ of 0.59 J that is calculated as

(5) $$E_{rot}=\frac{1}{2}I\times \omega^{2}$$

where $E_{rot}$ is the kinetic energy of rotation, $I_{bar}$ is the moment of inertia of the bar, $I_{sphere}$ is the moment of inertia of the sphere, and ω is the angular velocity of the pendulum.

The pendulum’s material, shape, and size were selected to obtain an impact force of 5N at a known velocity. This value was chosen as representative of the upper strength limit of the built neck dummy prototype, in order to obtain the most significant impact possible without damaging the system. The aim of the test is to verify the mobility and response of the vertebral unit to an impact through evaluating displacements, forces, and accelerations.

Figure 7a shows a CAD design of the neck prototype placed under the pendulum in the initial phase. Figure 7b shows the moment when the pendulum hits the head supported by the neck. At the moment of impact, the head and each vertebra suffer an acceleration due to the force of the impact.

### 2.6. Experimental Setup

**Figure 8:**
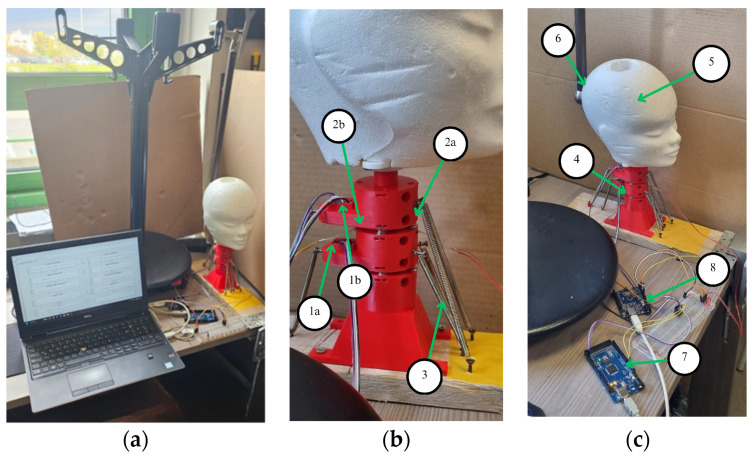
Experimental setup for lab testing: (**a**) view of the experimental setup; (**b**) sensors and springs; (**c**) prototype for a test. (The numbered elements are described in Table 5).

Figure 8 shows the prototype in the experiment setup, which consists of the two-vertebra units with vertebrae V1 and V2 that are connected through springs and flexible coupling. The prototype is fixed on a base frame representing the thoracic vertebra. A head is attached at the top of the neck with an added mass of 250 g. In addition, tension springs are attached to the front and rear part of each vertebra to replicate the action of the muscles.

The data acquisition system consists of an Arduino UNO microcontroller that receives data from the two BMI 160 sensors that are located on each vertebra working at a frequency of 50 Hz, while the force sensors on the front and back of the vertebra are connected to an additional Arduino Mega 2560 microcontroller. The data acquired from these microcontrollers are stored in a PC where the angles and injury index are computed. The main parts shown in Figure 8 are listed in Table 5.

**Figure 9:**
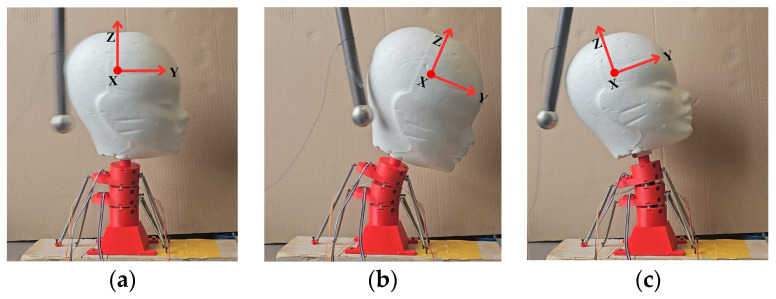
Snapshot of a test: (**a**) moment of impact to the head; (**b**) maximum forward displacement of the head; (**c**) maximum backward displacement of the head.

Figure 9 shows snapshots of the motion of the neck during a test in three important instants. The instant when the pendulum impacts the head is shown in Figure 9a. The instant with largest frontal displacement due to the impact is shown in Figure 9b. The instant of the largest reaction backwards is shown in Figure 9c.

## 3. Results

**Figure 10:**
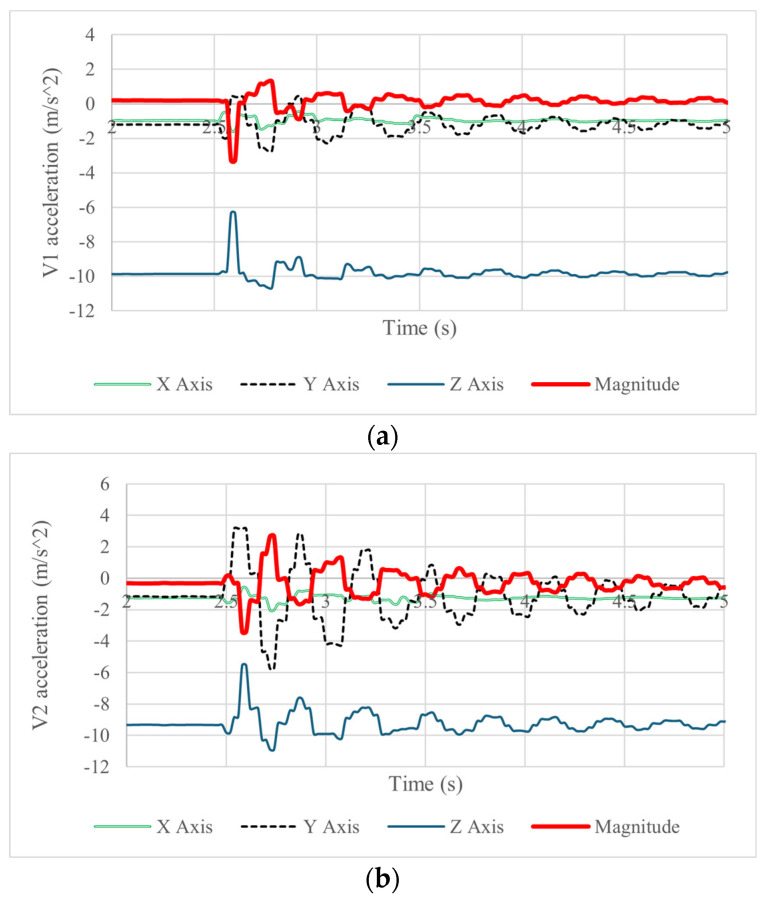
Acquired results of a test like in Figure 9 in terms of acceleration of (**a**) vertebra 1 and (**b**) vertebra 2.

**Figure 11:**
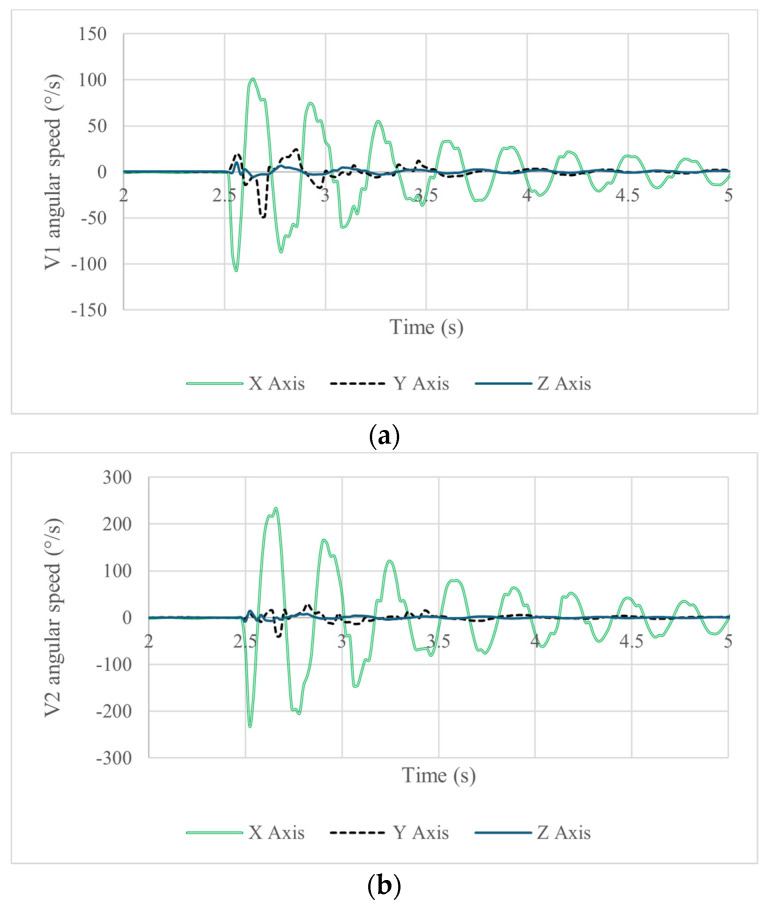
Acquired results of a test like in Figure 9 in terms of angular speed of (**a**) vertebra 1 and (**b**) vertebra.

**Figure 12:**
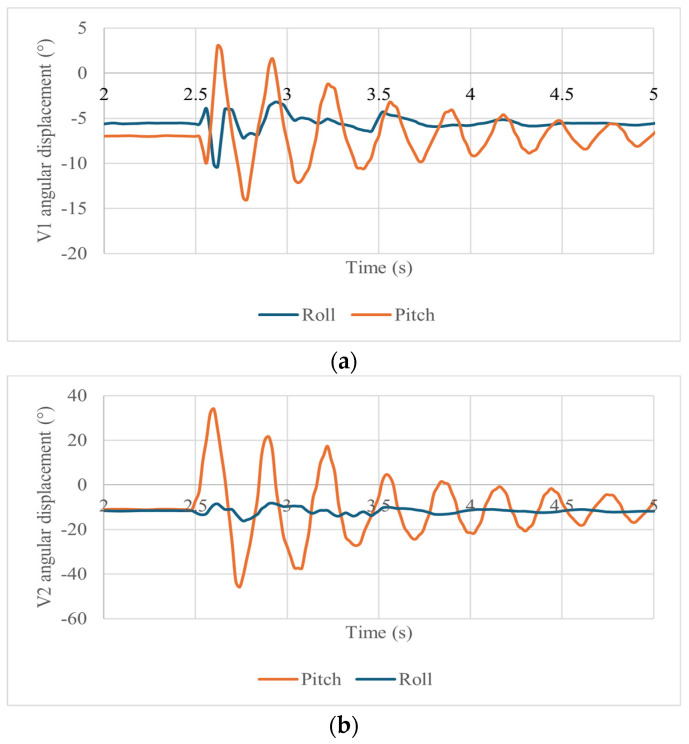
Computed angular Roll and Pitch displacements during a test like in Figure 9 for (**a**) vertebra 1 and (**b**) vertebra 2.

**Figure 13:**
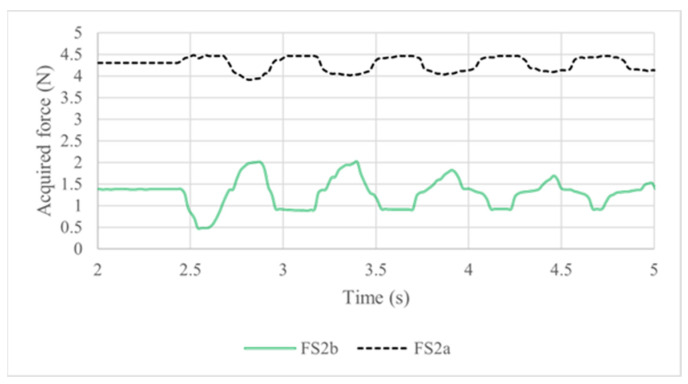
Results acquired during a test like in Figure 9 in terms of force sensed by force sensors.

**Figure 14:**
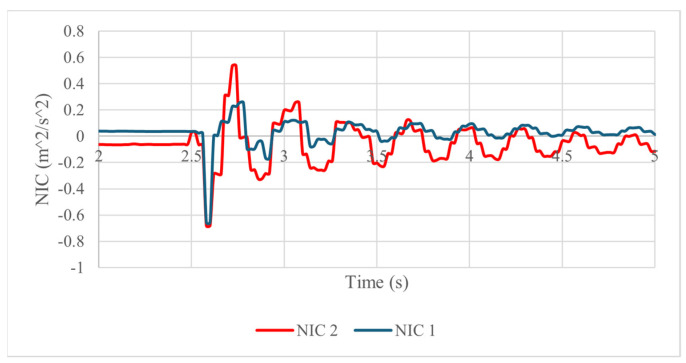
Computed results in terms of NIC for vertebra 1 and vertebra 2.

A test like the one in Figure 8 and Figure 9 was repeated five times to obtain a statistical significance of the results with a satisfactory repeated output. Illustrative results are reported in Figure 10, Figure 11, Figure 12, Figure 13 and Figure 14 and Table 6, Table 7 and Table 8. The acquired results from IMU sensors in terms of acceleration, angular speed, and angular displacement are shown in Figure 10, Figure 11 and Figure 12.

Figure 10a,b show the acquired accelerations of the vertebrae’s motion during the forward and backward motion of the neck with a maximum acceleration magnitude of 3.7 $m/s^{2}$ in vertebra 1 and 3.8 $m/s^{2}$ in vertebra 2. The flexion–extension of the neck is mainly detected by the Y and Z components of the acceleration, which in vertebra 1 shows maximum values of forward acceleration of 2.5 $m/s^{2}$ and backward acceleration of 2.3 $m/s^{2}$. This behavior is also detected in vertebra 2 but with a forward acceleration of 4.2 $m/s^{2}$ and a backward acceleration of −$6\;m/s^{2}$ in the Y component. The Z component of acceleration presents a maximum range of acceleration of 4 $m/s^{2}$ in both vertebrae. The decrease in the acceleration amplitude with respect to time shows that the damping in the system is low since the values oscillate significantly for 5 s. The results in the plots also show that the flexible joint and the tension springs can resist an impact and bring the prototype back to its original position, but an adequate damping element should have produced better behavior.

Figure 11 shows the acquired data in terms of angular velocity. The axis of main rotation is the X-axis due to the direction of impact shown in Figure 11. Like in the acceleration plots, one can observe a difference in the acquired magnitudes between the data for vertebra 1 and vertebra 2. The maximum angular speed in vertebra 1 is 101°/s forward and 106.5°/s backward. Vertebra 2 experienced an angular speed of 232.6°/s forward and 229.6°/s backward, indicating that both vertebrae respond at the same time to the given disturbance to the head.

Using the accelerometer and gyroscope data, the angular displacement of the vertebrae can be determined. Since the accelerometer data are a measure of the rotated gravity field vector, they can be used to determine the accelerometer angle and Roll orientation angles with the expressions

(6) $$\zeta =\tan^{-1}\left(\frac{a_{x}}{\sqrt{a_{y}^{2}}+a_{z}^{2}}\right)*\pi$$

(7) $$\theta =\tan^{-1}\left(\frac{a_{y}}{\sqrt{a_{x}^{2}}+a_{z}^{2}}\right)*\pi$$

where ζ and θ are the Pitch and Roll angles, respectively, and $a_{x}$, $a_{y}$, and $a_{z}$ are the components of the acceleration acquired by the IMU sensor. In addition, the orientation angle of the IMU can be obtained from the gyroscope signal by numerically integrating the angular velocity with respect to the time. To combine these measurements for Roll angle data, a complementary filter can be used to avoid low-frequency error from the gyroscope and high-frequency error from the accelerometer using the expression [41]

*θ*_I_ = VD × (*θi* − 1 + *ω* × *dt*) + AD × (*θacc*) (8)

where $\theta_{i}$ is the filtered measured angle; (*θi* − 1 + *ω*∗*dt*) is the computed angle using the gyroscope; *θacc* is the computed angle using the acceleration components; VD is percentage of the data use from the gyroscope; and AD is percentage of the data use from the accelerometer (the sum of VD and AD must be equal to one). For the correct work of the filter, we assumed 0.98 for the VD and 0.02 for the AD so that the computed angle from the accelerometer is elaborated through a low-pass filter, damping sudden variations in acceleration, and the angle calculated from the gyroscope data is tuned with a high-pass filter that is convenient when there are fast rotations. The resultant angular displacements are shown in Figure 12a for vertebra 1 and in Figure 12b for vertebra 2.

Figure 12a,b show the angular displacements of the vertebrae in terms of the Pitch and Roll angles. In Figure 12a, vertebra 1 shows a maximum angular displacement of 4° forward and 14° backward. In Figure 12b, vertebra 2 has a maximum forward displacement of 32° and a maximum backward angular displacement of 43°. In both cases, oscillations occur with quite the same frequency until they stop. The movement of both vertebrae is detected with a Roll component, indicating that the prototype does not move only in the direction of impact. This is most evident in vertebra 1, Figure 12a, which after impact has a Roll of 14°. This value is initially larger than the Pitch but is considerably reduced in later instants.

The results from force sensors 2a and 2b located in vertebra 1, Figure 8, indicated as FS2a and FS2b, were monitored simultaneously to the IMU sensors. Considering the initial position of the prototype with a slight deviation to the front and the applied pressure due to the tension springs, Figure 13 shows the initial condition with a 4.3 N preload in the FS 2a sensor and 1.3 N in the FS 2b sensor. When the pendulum impacts the back of the head, the FS 2a sensor value has only a slight increase to 0.6 N. When the prototype moves backwards, the load in the FS2a sensor value decreases to 4.1 N. The FS2b sensor detects a large force range from 0.6 N to 2.05 N.

The resulting torque on vertebra 1 from the force sensed by the force sensors FS2a and FS2b as coming from the metal springs and the flexible coupling can be calculated referring to the effect on the flexion and extension of the neck through MS2a and MS2b, respectively, as

MS2a = FS2a (d/2) and MS2b = FS2b (d/2)(9)

The acquired forces give a maximum torque of 0.15 Nm in neck extension and flexion as related to the largest angular displacements forward and backward. The most significant results are summarized in Table 6.

The results of all the tests demonstrate the feasibility of the proposed design and of the experiments conducted since they show that the prototype has the ability to replicate the neck’s response to an impact with results that are useful to predict the risk of injury. With the acquired data from the IMU sensors and force sensors, two neck injury criteria are computed to check the feasibility of the designed neck dummy and the testing layout in representing neck–head motion in impact with injury risk. The results of the NIC index using Equation (2) are summarized in Table 7 and represented in Figure 14 with the NIC calculated for both vertebrae. Similarly, the results for the Nij index using Equation (1) are summarized in Table 8.

The NIC computation results in Figure 14 show a bigger injury probability when the acceleration and the speed of the vertebra are large at the beginning of the impact response, as in Figure 10 and Figure 11. The summarized results of the five tests in Table 7 show a bigger probability of injury in the second vertebra since vertebra 2 experiences larger angular displacement and accelerations than vertebra 1. Nevertheless, the energy applied to the prototype is not enough to reach the 15 $\frac{m^{2}}{s^{2}}$ which is the proposed injury threshold of the NIC reported in [42].

The computed values of Nij summarized in Table 8 show the results of the injury index with a small deviation among tests. However, the force and torque values are far from the critical injury values mentioned in Table 4 [36]. The variation in forces and torque among the five tests is limited, probably due to the stiffness of the springs and the impact energy.

The maximum value of the NIC is obtained just moments after the impact, when there is a large acceleration in the vertebrae. Because of this, the calculated NIC is always larger in vertebra 2 than in vertebra 1 since it is vertebra 2 that experiences more acceleration after the impact of the pendulum. The maximum value of Nij is computed when both vertebrae have a large angular displacement, corresponding to the situation in which the forces acting on the springs sensed by force sensors are large. The reported results show that the response of the designed vertebral unit to an impact to the head can replicate the behavior of a human neck well. Furthermore, the results of the test show the ability of the model to acquire data that are useful for calculating injury criteria by considering each individual vertebra. The springs’ arrangement is able to replicate the muscle structure and flexibility of the vertebral unit. However, further analysis of the relationship of spring stiffness to neck response and a damping system should be implemented in the future to replicate the soft tissue behavior of the neck.

## 4. Discussion

The results of the experiments demonstrate the prototype’s ability to respond to external disturbances, simulating the neck’s reactive behavior against impacts well. The data acquisition system is aimed at monitoring the acceleration and force responses in the model, which are necessary to evaluate the probability of neck injury. The location of the force sensors on the vertebra unit provides an interesting perspective of the kinematic behavior and the distribution of loads on the cervical spine. Together with the implementation of IMU sensors on each vertebra, it is possible to relate the linear acceleration and the angular displacement of each vertebra to the forces acting on the intervertebral disc. The proposed two-vertebra unit with sensors gives more information than the existing solutions that were developed mainly for the evaluation of the injury risk indices. In fact, the two-vertebra unit is also intended to simulate and understand the effect of an impact of on a vertebra before considering the full cervical spine.

On the other hand, the use of a pendulum for laboratory tests limits the scope of the experimental investigation in simulating impacts, mainly from car accidents. The direct blow to the head used gives results that cannot permit a proper comparison of the results from studies considering vehicular impact conditions, but it does allow the evaluation of the flexibility of the neck dummy and its response to a disturbance from impacts. For this reason, the further development of laboratory tests is planned to simulate the conditions of vehicular impacts. In addition, the prototype design should be revised and expanded to a full neck with seven cervical vertebrae.

## 5. Conclusions

The design of a vertebra unit is presented with the features and functioning of a cervical spine in simulating the neck’s response to impacts. Problems have been discussed in identifying a central role of the neck in assessing injury risk in head impacts that are usually considered for car accidents. The built prototype of a two-vertebra unit at a large scale has been successfully used in tests both to validate the proposed design with its peculiar architecture using flexible joints and springs and to characterize the impact response for an injury risk evaluation. The proposed cervical vertebra unit will be the base for future development in designing a full-cervical-spine neck dummy. The good reported results show that the proposed prototype is able to react to external perturbations and provide useful data for calculating neck injury rates and give a better understanding of what happens in each vertebra individually. These results also validate the proposed novel design for a neck dummy, which will be expanded in future works to include seven vertebrae.

## Figures and Tables

**Table 1:** The average range of motion between each cervical vertebra [26].

	C0–C1	C1–C2	C2–C3	C3–C4	C4–C5	C5–C6	C6–C7
Flexion	7.2°	12.3°	3.5°	4.3°	5.3°	5.5°	3.7°
Extension	20.2°	12.1°	2.7°	3.4°	4.8°	4.4°	3.4°
Axial rotation	9.9°	56.7°	3.3°	5.1°	6.8°	5.1°	2.9°
Lateral bending	9.1°	6.6°	8.6°	9.0°	9.3°	6.5°	5.4°

**Table 2:** Intervertebral discs’ dimensions and mechanical properties [27].

Disc	Area $\left({mm}^{2}\right)$	Height (mm)	Load (N)	Def (mm)	E (Mpa)	Stiffness (N/mm)
C2-C3	162–369	3.8–5.8	602	1.4	10.09	637.5
C3-C4	153–561	4.5–6	683	1.5	13.39	765.3
C4-C5	151–365	4.6–6.5	777	1.6	14.79	784.6
C5-C6	194–588	4.2–7.2	664	1.6	8.98	800.2
C6-C7	307–753	5–7.5	673	1.7	6.45	829.7
C7-T1	309–715	4.5–67.2	910	1.6	8.28	973.6

**Table 3:** Dimensions and mass of the 50th percentile male head.

Anthropomorphic Measurement	Value
Head length	19.86 cm
Head breadth	15.57 cm
Head-to-chin height	23.24 cm
Head weight	3.9 to 4.6 kg

**Table 4:** Critical values in main criteria for injury risk.

Criterion	Parameter	Values
$N_{ij}$ [34]	Compression	4500 N
	Tension	4500 N
	Flexion	310 Nm
	Extension	125 Nm
NIC [40]	Human tolerance level	$<15\frac{m^{2}}{s^{2}}\;\;$
$N_{km}$ [36]	Sheer force	845 N
	Flexion	88.1 Nm
	Extension	47.5 Nm

**Table 5:** Main parts of the neck dummy testing setup.

Number	Description
1a and 1b	V1 BMI 160 and V2 BMI 160
2a and 2b	Frontal FSR 402 and Rear FSR 402
3	Tension springs
4	Neck prototype
5	250 g head
6	Pendulum
7	Arduino Mega 2560
8	Arduino Uno

**Table 6:** A summary of results from Figure 10, Figure 11, Figure 12 and Figure 13.

Test No.	$a_{1}$ $\left(m/s^{2}\right)$	$a_{2}$ $\left(m/s^{2}\right)$	$v_{1}$ $\left(m/s\right)$	$v_{2}$ $\left(m/s\right)$	FS2a (N)	MS2a (Nm)	FS2b (N)	MS2b (Nm)
1	2.1	4.2	0.005	0.02	5	0.2	−1.9	0.05
2	1.9	4.9	0.0046	0.021	4.8	0.2	−2	0.1
3	1.9	4.9	0.0038	0.021	4.5	0.2	−2.1	0.09
4	1.1	5.1	0.0051	0.019	5	0.21	−2.1	0.09
5	1.2	4.9	0.0041	0.021	5	0.2	−1.6	0.09

**Table 7:** Acquired results from test with values of NIC computed with Equation (2).

Test No.	$a_{1}$ $\left(m/s^{2}\right)$	$a_{2}$ $\left(m/s^{2}\right)$	$v_{1}$ (m/s)	$v_{2}$ (m/s)	V_1_ NIC ${(m}^{2}/s^{2})$	V_2_ NIC ${(m}^{2}/s^{2})$
1	2.1	4.2	0.005	0.02	0.45	0.49
2	1.9	4.9	0.0046	0.021	0.38	0.6
3	1.9	4.9	0.0038	0.021	0.29	0.43
4	1.1	5.1	0.0051	0.019	0.20	0.21
5	1.2	4.9	0.0041	0.021	0.21	0.5

**Table 8:** Acquired results from test with values of Nij computed with Equation (1).

Test No.	F1 (N)	M1 (Nm)	F2 (N)	M2 (Nm)	Flexion Nij	Extension Nij
1	5	0.2	−1.9	0.05	$17\times 10^{-4}$	$2\times 10^{-3}$
2	4.8	0.2	−2	0.1	$17\times 10^{-4}$	$1.2\times 10^{-3}$
3	4.5	0.2	−2.1	0.09	$16\times 10^{-4}$	$1.1\times 10^{-3}$
4	5	0.21	−2.1	0.09	$17\times 10^{-4}$	$1.1\times 10^{-3}$
5	5	0.2	−1.6	0.09	$17\times 10^{-4}$	$1\times 10^{-3}$

## Data Availability

The original contributions presented in this study are included in this article; further inquiries can be directed to the corresponding author.

## References

[1] World Health Organization Global Status Report on Road Safety 2023. https://www.who.int/data#reports.

[2] Quinlan K.P., Annest J.L., Myers B., Ryan G., Hill H. (2004). Neck strains and sprains among motor vehicle occupants—United States, 2000. Accid Anal. Prev.

[3] Panjabi M.M., Cholewicki J., Nibu K., Grauer J.N., Babat L.B., Dvorak J. (1998). Mechanism of whiplash injury. Clin. Biomech..

[4] Stemper B.D., Yoganandan N., Pintar F.A. (2004). Gender- and Region-Dependent Local Facet Joint Kinematics in Rear Impact. Spine (Phila Pa 1976).

[5] Bumberger R., Acar M., Bouazza-Marouf K. (2020). Importance of intervertebral displacement for whiplash investigations. Int. J. Crashworthiness.

[6] Cormier J., Gwin L., Reinhart L., Wood R., Bain C. (2018). A comprehensive review of low-speed rear impact volunteer studies and a comparison to real-world outcomes. Spine.

[7] Bogduk N., Yoganandan N. (2001). Biomechanics of the cervical spine Part 3: Minor injuries. Clin. Biomech..

[8] Kang Y.-S., Millis W., Thomas C., Stricklin J., Willis A., Pradhan V., Tesny A., Kwon H.J., Ramachandra R., Moorhouse K. Head-to-T1 Relative Rotation of the BioRID-II, Hybrid III, and Post Mortem Human Subjects with Increased Backsets in Moderate-Speed Rear Impacts. Proceedings of the 2021 IRCOBI Conference Proceedings.

[9] Ivancic P.C., Ito S., Panjabi M.M., Pearson A.M., Tominaga Y., Wang J.L., Gimenez S.E. (2005). Intervertebral Neck Injury Criterion for simulated frontal impacts. Traffic Inj. Prev..

[10] Alpini D.C., Brugnoni G., Cesarani A. (2014). Whiplash Injuries.

[11] Hong L., Liu G. (2021). Analysis on the driver’s upper and lower neck injury in the three stages of the frontal collision. Proc. Inst. Mech. Eng. Part. D J. Automob. Eng..

[12] Beeman S.M., Kemper A.R., Duma S.M. (2016). Neck forces and moments of human volunteers and post mortem human surrogates in low-speed frontal sled tests. Traffic Inj. Prev..

[13] Garrosa M., Ceccarelli M., Díaz V., Russo M. (2023). Experimental Validation of a Driver Monitoring System. Machines.

[14] Mohan P., Marzougui D., Kan C.-D. (2009). Development and Validation of Hybrid III Crash Test Dummy.

[15] Somers J.T., Newby N., Lawrence C., DeWeese R., Moorcroft D., Phelps S. (2014). Investigation of the THOR anthropomorphic test device for predicting occupant injuries during spacecraft launch aborts and landing. Front. Bioeng. Biotechnol..

[16] Rueda-Arreguín J.L., Ceccarelli M., Torres-SanMiguel C.R. (2022). Design of an Articulated Neck to Assess Impact Head-Neck Injuries. Life.

[17] Wang Y., Jiang H., Teo E.C., Gu Y. (2023). Finite Element Analysis of Head–Neck Kinematics in Rear-End Impact Conditions with Headrest. Bioengineering.

[18] Henriques D., Martins A.P., Carvalho M.S. (2024). Efficient 2D Neck Model for Simulation of the Whiplash Injury Mechanism. Bioengineering.

[19] Xu T., Sheng X., Zhang T., Liu H., Liang X., Ding A. (2018). Development and Validation of Dummies and Human Models Used in Crash Test. Appl. Bionics Biomech..

[20] Albert D.L. A Comparison of Two Versions of the Biofidelity Ranking System (BioRank): Implications on THOR- 50M and THOR-05F Biofidelity Evaluations. Proceedings of the 2020 IRCOBI Conference Proceedings.

[21] MacGillivray S., Wynn G., Ogle M., Shore J., Carey J.P., Dennison C.R. (2021). Repeatability and Biofidelity of a Physical Surrogate Neck Model Fit to a Hybrid III Head. Ann. Biomed. Eng..

[22] Rhule H.H., Maltese M.R., Donnelly B.R., Eppinger R.H., Brunner J.K., Bolte J.H. Development of a New Biofidelity Ranking System for Anthropomorphic Test Devices. Proceedings of the 46th Stapp Car Crash Conference.

[23] Viano D.C., Davidsson J. (2002). Neck displacements of volunteers, BioRID P3 and Hybrid III in rear impacts: Implications to whiplash assessment by a Neck Displacement Criterion (NDC). Traffic Inj. Prev..

[24] D’Arienzo M., Peri G., Valentino B., Conti A., D’Arienzo A., Peri D. (2016). Anatomy of the Cervical Spine. Cervical Spine.

[25] Ramieri A., Domenicucci M., Miscusi M., Costanzo G. (2016). Functional Anatomy and Biomechanics of the Cervical Spine. Cervical Spine.

[26] Panjabi M.M., Crisco J.J., Vasavada A., Oda T., Cholewicki J., Nibu K., Shin E. (2001). Mechanical Properties of the Human Cervical Spine as Shown by Three-Dimensional Load–Displacement Curves. Spine (Phila Pa 1976).

[27] Yoganandan N., Kumaresan S., Pintar F.A. (2001). Biomechanics of the cervical spine Part 2. Cervical spine soft tissue responses and biomechanical modeling. Clin. Biomech..

[28] Yoganandan N., Maiman D.J., Guan Y., Pintar F. (2009). Importance of physical properties of the human head on head-neck injury metrics. Traffic Inj. Prev..

[29] Yoganandan N., Pintar F.A., Zhang J., Baisden J.L. (2009). Physical properties of the human head: Mass, center of gravity and moment of inertia. J. Biomech..

[30] Foster J.K., Kortge J.O., Wolanin M.J. Hybrid III-A Biomechanically-Based Crash Test Dummy. Proceedings of the 21st Stapp Car Crash Conference.

[31] Haffner M., Rangarajan N., Artis M., Beach D., Eppinger R., Shams T. Foundations and Elements of the NHTSA THOR Alpha ATD Design. Proceedings of the 17th ESV Conference.

[32] Albert D.L., Beeman S.M., Kemper A.R. (2018). Evaluation of Hybrid III and THOR-M neck kinetics and injury risk under various restraint conditions during full-scale frontal sled tests. Traffic Inj. Prev..

[33] Rhule H., Moorhouse K., Donnelly B., Stricklin J. Comparison of WorldSID and ES-2RE biofidelity using an updated biofidelity ranking system. Proceedings of the 21st (ESV) International Technical Conference on the Enhanced Safety of Vehicles.

[34] Matsui Y., Kubota M., Oikawa S. (2018). Similarity of the measured NIC of a BioRID II dummy in car to car rear end impact and sled test experiments. Int. J. Automot. Technol..

[35] Simms C., Wood D. (2009). Injury Mechanisms and Injury Criteria. Pedestrian and Cyclist Impact. Solid Mechanics and Its Applications.

[36] Eppinger R., Sun E., Bandak F., Haffner M., Khaewpong N., Maltese M., Kuppa S., Nguyen T., Takhounts E., Tannous R. (1999). Development of Improved Injury Criteria for the Assessment of Advanced Automotive Restraint Systems-II.

[37] Schmitt K., Muser M.H., Niederer P. (2001). A New Neck Injury Criterion Candidate for Rear-End Collisions Taking into Account Shear Forces and Bending Moments.

[38] Schmitt K.U., Muser M.H., Walz F.H., Niederer P.F. (2002). Nkm—A proposal for a neck protection criterion for low-speed rear-end impacts. Traffic Inj. Prev..

[39] Garrosa M., Ceccarelli M., Díaz V. (2022). Problems and Requirements in Impact Analysis from Vehicle Accidents. Mechanisms and Machine Science.

[40] Croft A.C., Herring P., Freeman M.D., Haneline M.T. (2002). The neck injury criterion: Future considerations. Accid. Anal. Prev..

[41] Montero F.E., Arencibia G., Álvarez J., Suarez J.R., Molinet A.P. (2020). Estimación de orientación, basada en filtro de Kalman, usando unidad de medición inercial sin magnetómetro. Investig. Oper..

[42] Panjabi M.M., Ito S., Ivancic P.C., Rubin W. (2005). Evaluation of the intervertebral neck injury criterion using simulated rear impacts. J. Biomech..

